# Observations on Poisons; and on the Use of Mercury in the Cure of Obstinate Dysenteries

**Published:** 1784

**Authors:** 


IV. *Obſervations on Poiſons; and on the uſe of Mercury in the cure of obſtinate Dyſenteries. By* Thomas Houlſton, M. D. *Phyſician to the Liverpool Infirmary, and Honorary Member of the Literary and Philoſophical Society of Mancheſter.* 8vo. Baldwin, *London*, 1784. 72 pages, 1s.

THE obſervations on mineral poiſons—the caſe of a boy poiſoned by the roots of the the hemlock dropwort*—the directions for giving affiftance to perfons dying from drinking fpirits—and the obfervations on canine madnefs, which form the moft confiderable part of this little work, have already appeared in different periodical publications, and are here collected and re-printed, with an Introduction, in which the author gives a fummary but judicious account of the feveral poifons, and of the means of counteracting their effects. An engraving is added of the *Bunium-bulbocaftanum* and *Oenanthe-crocata*.

The papers on poifons are followed by a very interefting account, not before publifhed, of the good effects produced by mercurials in three cafes of obftinate dyfentery, which feemed to depend on a difeafe of the liver.

The fubject of the firft of thefe cafes was a feaman, who had been two years on the coaft of Africa, where he had an intermittent fever of long duration, and who was admitted into the Liverpool infirmary for the cure of a dyfentery, which he had laboured under for two years.—He was a ftout-made man, about fifty-eight years of age, but had a very fallow complexion, and a prominent belly, the region of the liver being enlarged, and, on preffure, painful.

* See the 2d vol. of this Journal, page 40.

In

In this caſe, after the moſt probable and uſual means of putting a ſtop to the dyſentery had been purſued for the ſpace of eight months, without any conſiderable good effect, our author began to reflect, that the dyſentery might very probably originate from a diſeaſed liver; and that if this were the caſe, it was not likely that the flux ſhould be got the better of, unleſs the affection of the liver on which it depended was firſt removed. With this view, he directed the mercurial inunctions to be gradually applied, and, as no increaſe of the dyſenteric ſymptoms followed their uſe, they were continued (a fortnight) till the mouth was affected, and a moderate ſalivation came on. When this took place, his ſtools became leſs frequent, more regular, natural, and free from blood. By the time it had ceaſed, he thought himſelf freed from all his complaints, and, at his own requeſt, was diſcharged.

A fortnight after, he applied again for admiſſion: his appetite was impaired, his gripings violent, his ſtools very frequent and bloody; and his belly, about the region of the liver, was ſwelled, hard, and painful. After premiſing a few gentle evacuants, the inunctions were repeated, peated. For ſome days he was no better, and, being rather feveriſh, the mercury was omitted for a week, and then reſumed, by which means a ptyaliſm was produced. He was then very eaſy in his belly, his looſeneſs was almoſt ſtopped, and he ſaid himſelf he was much better than ever he had been ſince the beginning of his illneſs. The mercurials, after a little reſpite, were continued ſome time longer, and he was then diſcharged perfectly well, and ſo remained.

The ſecond patient was likewiſe a mariner, 28 years old, of a ſallow, bilious complexion, who had paſſed much of his life in hot climates, and who was admitted into the infirmary Sept. 23, 1779, for a dyſentery of ſix years ſtanding. As this man had been in different hoſpitals in London and other places, Dr. Houlſton thought it unneceſſary to attempt to ſucceed in his cure by the uſual remedies, and therefore determined to try the effect of mercurials.

The patient was directed to rub in half a drachm of ſtrong mercurial ointment every other evening; which he continued to do till October 9, when a ptyaliſm was produced, which continued in a conſiderable degree ten

 or or twelve days. During this time he took only the Decoctum Album, and Caſtile Soap. In three days after the ſpitting began, his flux ſtopped, and his ſtools, of which he had not more than one or two in twenty-four hours, were natural, and without any griping. He had, however, a very acute head-ach, which gradually went off, and by the end of the month he could, without inconvenience, eat broths, and other things, which before this time uſed to render his complaint violent.

Still, however, the purging returned at times ſoon after, though not with the former violence. At his own requeſt, therefore, he began again with the inunctions, Nov. 25, which excited ſalivation in leſs than a fortnight, and ſeemed to have carried off the complaint; but as the ſtomach and inteſtines were greatly debilitated, our author gave him, at different times, the ſal martis, bark, and other aſtringents. Towards the end of January, 1780, he was attacked with a rheumatic complaint, which he abſcribed to cold from changing his room, but which yielded ſoon to the Decoctum Guaici. About the middle of February, he was attacked with a ſlight tertian ague, to which he had been ſubject before, but which went off in a few few days. In the beginning of March he was free from both, and having ſignified a deſire of going to ſea, was diſcharged from the infirmary.

The account he then gave of himſelf was as follows; "Of ſtools he had two or three in twenty-four hours, eaſy and natural, ſometimes more coſtive than he wiſhed, on account of his hœmorrhoids. Perhaps once in a fortnight, he had a purging, which continued about twenty-four hours. His appetite was poor, but what he eat, (in which he was not very cautious) ſat eaſier upon his ſtomach, and agreed better with him, than it had uſed to do, and his health and ſtrength were much better than at any time ſince his diſorder began."

The third patient was a German ſailor, 22 years of age, who was admitted into the Liverpool infirmary for a dyſentery, which began during his paſſage from Jamaica, and had continued about three months. In this caſe, our author, after having tried a variety of evacuant and aſtringent medicines for three months, with but very little advantage, and that not permanent, had recourſe to a mercurial courſe. Half a drachm of the ſtrongeſt mercurial ointment was rubbed in every night for a month, when it was diſcontinued, on account of a tertian ague, which yielded to an emetic before the cold fit, and an opiate in the beginning of the hot one. No ſalivation had taken place, but his ſtools were regular, without pain or blood, and not more than two in twenty-four hours; ſo that he left the infirmary, and returned to his uſual employment ſeemingly in perfect health.

Theſe are the only caſes, of which our author has preſerved the particulars; but, it ſeems, that in ſeveral other caſes of a ſimilar nature, he has found the ſame mode of treatment equally ſucceſsful.

Towards the concluſion of his work, Dr. Houlſton offers ſome remarks on the dry vomit, recommended by the late Dr. Maryatt, which is a compoſition of equal parts of emetic tartar and blue vitriol. The doſe of this mixture, commonly given, is five grains, on an empty ſtomach, in about half a table ſpoonful of water. Dr. Houlſton has given it in a variety of caſes, and it has acted ſo mildly, that he ſcarcely recollects an inſtance where it has been complained of as too violent; but he has met with ſeveral wherein five grains were not ſufficient to produce any effect, and where he has has found it neceſſary to increaſe the doſe to ſeven or eight grains. The reaſon, he obſerves, why the compound acts more mildly than one of the ingredients would do alone, is not eaſy to aſcertain. But it is ſufficient, he adds, for medical purpoſes to know, that it is not only a ſafe but even a mild vomit; and he thinks, that in caſes where we wiſh to evacuate bile, or to give a ſtimulus to the whole ſyſtem, from the action of vomiting, the dry vomit will anſwer the ſame purpoſe as ſea ſickneſs.

Dr. Houlſton takes occaſion to give his teſtimony in favour of the method of cure recommended by Dr. Lind, in caſes of intermittents, viz. the giving a vomit an hour before the cold fit, and a ſufficient doſe of Tinct. Thebaic. half an hour after the hot fit commences. In many intermittents of long continuance, both tertians and quartans, our author has known this method put a ſtop to the diſeaſe the very firſt time it was made uſe of. But although this will often not be the caſe, and it will be neceſſary to repeat the Tinct. Thebaic. on each acceſſion of the hot fit, and to increaſe the doſe of it, yet the great relief and the gradual diminution it occaſions in the ſtrength of the fits, are, he obſerves, ſtrong inducements to perſevere vere in the ufe of the remedy, till the difeafe is compleatly removed. This mode of treatment, it feems, has in general been fo fuccefsful in the hands of our author, that he has very rarely had occafion to recur to the bark for the cure of agues, though he fometimes gives it after the complaint is removed, with a view to ftrengthen the habit.

